# Stabilization and Valorization of Beer Bagasse to Obtain Bioplastics

**DOI:** 10.3390/polym15081877

**Published:** 2023-04-14

**Authors:** Daniel Castro-Criado, Johar Amin Ahmed Abdullah, Alberto Romero, Mercedes Jiménez-Rosado

**Affiliations:** Departamento de Ingeniería Química, Universidad de Sevilla, 41012 Sevilla, Spain

**Keywords:** bagasse, stabilization, valorization, bioplastics, circular economy

## Abstract

Beer bagasse is a residue produced in large quantities, though it is undervalued in the industry. Its high protein and polysaccharide content make it attractive for use in sectors such as the manufacture of bioplastics. However, its high water content makes it necessary to stabilize it before being considered as a raw material. The main objective of this work was to evaluate the stabilization of beer bagasse and the production of bioplastics from it. In this sense, different drying methods (freeze-drying and heat treatment at 45 and 105 °C) were studied. The bagasse was also characterized physicochemically to evaluate its potential. In addition, bagasse was used in combination with glycerol (plasticizer) to make bioplastics by injection molding, analyzing their mechanical properties, water absorption capacity and biodegradability. The results showed the great potential of bagasse, presenting a high content of proteins (18–20%) and polysaccharides (60–67%) after its stabilization, with freeze-drying being the most suitable method to avoid its denaturation. Bioplastics present appropriate properties for use in applications such as horticulture and agriculture.

## 1. Introduction

Currently, the economic model is linear; that is, it is based on the mentality of extracting, producing and discarding. Thus, a large number of raw materials and non-renewable energies are used in the manufacture, distribution and disposal processes, which generate a loss of resources and negative effects at the environmental and social level [1]. These drawbacks are raising awareness among people who try to reverse them. An example of this is the creation of the 2030 Agenda [2]. One of the established objectives is to guide the transition from the current linear economy model to a circular model. The efficient use of waste could have a direct impact on the economy and on environmental pollution. In this way, recovering residues could increase potential income by obtaining products with high added value, while reducing waste that must be treated. This can drive the industry towards sustainability, both economically and environmentally.

Beer is one of the most consumed beverages worldwide and an increase in consumer demand involves a gradual increase in its production. The increase in world production of beer presents an increase in fixed wastes generated during its production. The most abundant beer waste is bagasse (spent brewers’ grain), resulting from the filtering and pressing of the wort obtained after the saccharification of the malted barley grain [3]. It is estimated that, for each hectoliter of beer, approximately 20 kg of bagasse is obtained, which means a high availability of this waste. Normally, this waste is used as animal feed due to its low cost [4]. The world production of beer in 2021 was approximately 1.86 billion hectoliters, which represents around 37.2 billion tons of bagasse [5].

It is important to evaluate the chemical composition of bagasse to valorize it. Its composition is highly variable, mainly due to the selected production process, raw materials and the technological evolution of the sector. In this sense, it is important to take this compositional instability into account when valuing this raw material, requiring an exclusive study of each client. In general, it is estimated that the composition of beer bagasse, on a dry basis, is rich in proteins (15–25%) and lignocellulosic matter (70%), also presenting ashes (2–4.5%), lipids (3.9–18%) and extractable compounds, among which vitamins, amino acids and phenolic compounds stand out [6]. The lignin fraction contains numerous phenolic compounds such as ferulic, p-coumaric, syringic, vanillic and p-hydroxybenzoic acids [7]. It should be mentioned that the beer bagasse obtained industrially has high humidity (between 70 and 85%). This, added to the effect of the presence of lipids, protein matter and fermentable sugars, makes bagasse highly unstable, which can lead to the appearance of microorganisms and fungi that deteriorate its properties [8]. Thus, it is necessary to stabilize it by removing moisture.

There are several studies that try to give a new application to this beer bagasse to minimize its discharge as waste. Among them, Ortiz et al. tried to recover energy from beer bagasse to cover the energy requirement of the beer process, although this strategy did not seem profitable energetically nor economically in small breweries [9]. Morán-Aguilar et al. used it as raw material to produce xylanase and cellulase, although its scalability was low [10]. Texeira et al. produced volatile fatty acids (propionic, acetic and butyric acids) through anaerobic digestion using beer bagasse as feedstock [11].

The new alternative proposed in this work is the production of bioplastics. The concept of bioplastic can be understood from two different aspects: on the one hand, regarding its origin, which is natural, mainly from proteins and polysaccharides and, on the other hand, with respect to its biodegradability, which grants it the ability to be degraded under natural or composting conditions in short periods of time [12]. In this way, there are bioplastics from a non-natural origin which are biodegradable (i.e., polybutylene adipate terephthalate or polycaprolactone) [13] and, on the contrary, bioplastics from a natural origin which are not biodegradable (i.e., biobased polyethylene, polyethylene terephthalate or polyamide) [14]. In addition, there are bioplastics that meet both requirements, i.e., which come from natural materials and are biodegradable [15,16]. The latter are the most interesting, since they cover the two characteristics sought to replace conventional plastics. Nowadays, bagasse has been investigated as an additive to other polymers to improve their mechanical resistance [17,18]. Nevertheless, the composition of bagasse, mainly based on proteins and polysaccharides, grants it a high potential for the development of bioplastics that are of natural origin and biodegradable (without the need to use other polymers). All this can increase its value, becoming a profitable by-product instead of waste.

For this reason, the main objective of this work was to characterize and stabilize the bagasse from a brewery for use as raw material to produce bioplastics. The bagasse was characterized physicochemically and stabilized using two methods (freeze-drying and heat treatment at 45 and 105 °C). The different treatments were compared to determine the most suitable method and the bioactive compounds (proteins and polysaccharides) of the highest possible quality (without denaturing). Finally, its use as raw material to produce bioplastics was evaluated, using different mold temperatures (70, 90 and 110 °C) to shape them. The bioplastics were characterized to evaluate their mechanical properties, water absorption capacity and biodegradability. The novelty of this work was to carry out a complete study from the characterization of the raw material to the development of a final product with added value. This may be beneficial to show the potential of this waste and encourage further research in this field.

## 2. Materials and Methods

### 2.1. Materials

The beer bagasse used in this study was supplied by Heineken (Seville, Spain) and Guadalquibeer S.L. (Seville, Spain). This waste was collected from the company after beer production and without treatment; that is, with a high degree of humidity (>70%).

Glycerol, provided by Escuder (Barcelona, Spain), was used as plasticizer.

### 2.2. Bagasse Stabilization

The selection of the most suitable drying process depends on numerous factors (quantity of raw material, the final application of bagasse, amount of water present, etc.). There are three possible methods for reducing the water content in the bagasse: pressing, thermal treatment and freeze-drying. Pressing can reduce 60–70% of water content, which is not enough to stabilize the bagasse [19]. The other two methods (freeze-drying and thermal drying) are effective in eliminating at least 80% of the water present in the bagasse and can give a different quality to the raw materials obtained after stabilization. For this reason, they were the methods chosen in this study.

Freeze-drying was performed by freezing the sample at −40 °C for 2 h before undergoing the process in LyoQuest equipment (Telslar, Barcelona, Spain) at 0.1 mbar and −80 °C (chamber volume: 0.002 m^3^). Thermal drying was carried out in a JP Selecta oven (Barcelona, Spain) at 45 and 105 °C (chamber volume: 0.03 m^3^). The samples were tempered in a desiccator (volume: 0.01 m^3^) before being weighed. The drying kinetics of both methods were evaluated, as well as the quality of the product after their application. Drying was monitored over time by weighing the total sample. To avoid drying intervals, different samples were used, leaving each of them an opportune time necessary to evaluate the complete kinetics. The necessary drying time was established as that after which the weight of the sample did not change for at least 2 h.

Finally, all the samples were homogenized. Firstly, they were mechanically ground using a coffee grinder (Samsparty, Toledo, Spain) for 45 s. Then, they were manually sieved (mesh size of 420 µm). This sieved size was chosen because some authors associate the highest concentration of biopolymers with samples of smaller particle sizes, since the fibers tend to be larger after grinding [20].

### 2.3. Bioplastic Processing

Firstly, a dough-like blend was prepared using a 1:1 ratio of bagasse flour and glycerol. This ratio was selected to obtain a consistent and cohesive blend, avoiding both glycerol excess and blend granulation (see Results section). This homogenization was performed in a Haake Polylab QC batch mixer (Thermo Scientific, Dreieich, Germany) at 50 rpm for 10 min. The mixing time was evaluated to ensure an optimal mixing degree. For this, the torque and temperature were measured throughout the mixing.

The dough-like blend was subsequently processed by injection molding to obtain bioplastics. A MiniJet Piston Injection Molding System II (Thermo Scientific, Dreieich, Germany) was used with two different geometries: rectangular and dumbbell shape. The selected parameters were injection and holding pressure of 50 MPa (20 s) and 20 MPa (200 s), respectively, and cylinder temperature of 50 °C. Mold temperature was changed (70, 90 and 110 °C) to evaluate its influence.

**Figure 1:**
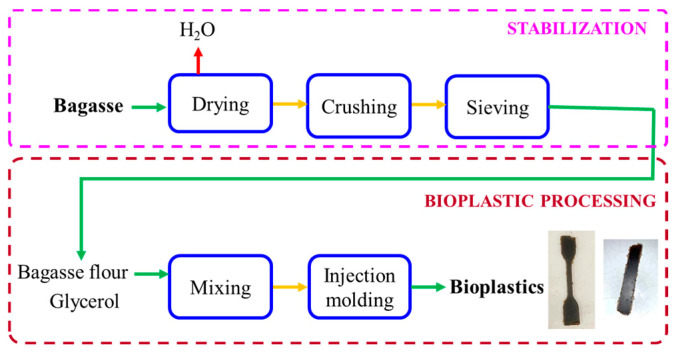
Scheme illustrating the process to obtain bioplastics from beer bagasse, with visual aspects of dumbbell and rectangular bioplastics.

Figure 1 shows the entire process from industrial beer bagasse to bioplastic.

### 2.4. Characterization Techniques

#### 2.4.1. Bagasse Characterization

The color of the samples was evaluated. For this, a Minolta 700 d spectrometer (Konica Minolta, Singapore) was used, where the values of the CIELAB uniform space were obtained. The luminosity, tone and chroma of the different samples were calculated following the protocol described by Jiménez-Rosado et al. [21].

The chemical composition of the initial and stabilized bagasse was obtained following A.O.A.C. methods [22]. Moisture was measured after subjecting the sample to a heat treatment at 105 °C in a JP Selecta oven (Spain) for 24 h. Ash content was obtained after calcining the volatile matter at 550 °C in an HD-240 furnace (Hobersal, Barcelona, Spain) for 5 h. A solid–liquid extraction was performed using the Soxhlet method to obtain the lipid content [23]. Proteins were obtained by elemental analysis in a LECO CHNS-932 nitrogen microanalyzer, applying a correction factor of 6.5. Finally, polysaccharide content was determined following the protocol used by He et al. [24]. Briefly, 0.5 g of sample was mixed with 0.5 mL of 5% phenol and 2.5 mL concentrated H_2_SO_4_ in an ice-water bath. Then, it was heated in boiling water for 15 min. The absorbance of the mixture was measured at 490 nm using a spectrophotometer Genesys-20 (Thermo Scientific, Waltham, MA, USA). A calibration curve was performed with sucrose (between 0 and 120 wt% concentration in sucrose with 15 points).

The chemical structures and interactions of the samples were also evaluated by Fourier Transform Infrared Spectroscopy (FITR). A Hyperion 100 spectrometer (Bruker, Billerica, MA, USA) with an ATR sensor was used. The measurements were obtained between 4000 and 500 cm^−1^ with an opening of 4 cm^−1^ and an acquisition of 200 scans.

Finally, the thermal transition of the samples was evaluated by scanning differential calorimetry (DSC). A DSC37 Q20 (TA Instruments, New Castle, DE, USA) with aluminum pan was used. The measurements were obtained for 0.2 g of sample between −20 and 120 °C using a heat rate of 10 °C/min.

#### 2.4.2. Blend Characterization

The blend was evaluated to optimize the injection process. To this end, its thermal profile was obtained in compression mode using a dynamic mechanical analyzer (DMA) 850 (TA Instruments, New Castle, DE, USA) with a plate–plate geometry (dia: 15 mm). The profile was obtained from 25 to 120 °C at a constant frequency of 1 Hz and a strain in the linear viscoelastic range (0.05%).

#### 2.4.3. Bioplastic Characterization

The mechanical properties of the bioplastics were measured in static and dynamic modes. Static mechanical tests were performed using Universal Insight 10 kN equipment (MTS, Eden Prairie, MN, USA) in tension mode. These tests were performed following ISO 527-2:2012 standard [25] at a strain rate of 1 mm/min. Young’s modulus, maximum stress and strain at break were obtained for each sample.

Dynamic mechanical tests were performed in a DMA RSA3 (TA Instrument, New Castle, DE, USA) using a dual cantilever geometry in flexural mode. Firstly, strain sweep tests were performed (between 0.002 and 2% at 1 Hz) to obtain the critical strain (last strain in the linear viscoelastic range). Strain sweep tests are shown in Figure S1. Then, frequency sweep tests were carried out (between 0.02 and 20 H at a constant strain (0.1% below the critical strain) to evaluate the stability of the bioplastics.

The water uptake capacity of these bioplastics was also obtained. For this, a rectangular bioplastic was immersed in 300 mL of distilled water for 24 h. Water uptake capacity was calculated following ASTM D570:1998 [26] through the difference in weight between the bioplastic before and after absorption. In addition, soluble matter loss was determined by comparing the dry weights before and after absorption.

Finally, biodegradation tests were performed following ISO 20200 standard [27]. For this, bioplastics were buried in a compostable medium (2:1 soil:compost with 10% vermiculite), maintaining a relative humidity of 80%. The biodegradation process was monitored visually and the biodegradation time was defined as the time after which no piece of bioplastic (>1 mm) was visible upon excavation.

### 2.5. Statistical Analysis

At least three replicates of each measurement were carried out. The values are represented as a mean value with its standard deviation, which was obtained using Excel software (Microsoft, Albuquerque, NM, USA). In addition, an analysis of variance (ANOVA) and a comparison of means with the Tukey method were carried out. The confidence level was set at 95% (*p* < 0.05).

## 3. Results and Discussion

### 3.1. Bagasse Stabilization

The water content of the bagasse after its industrial production is very high (>75%), as is shown in Table 1. In addition, this water has a high degree of activity since it is not bound to biological material. For this reason, the removal of this water is essential for the stabilization of the sample [28].

**Figure 2:**
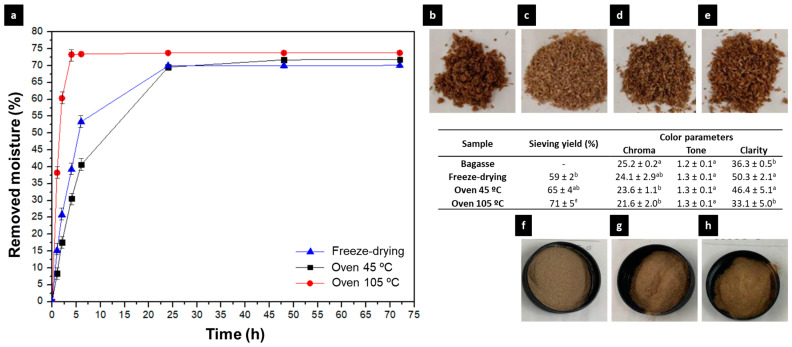
Bagasse stabilization. Kinetic profile of water removal with the different techniques (**a**). The appearance of raw bagasse (**b**) and stabilized bagasse after freeze-drying, oven 45 °C and oven 105 °C ((**c**–**e**), respectively). Color parameters of the different samples (Table). Different letters in the same column mean significant differences (*p* < 0.05). The visual aspect of stabilized bagasse after crushing/sieving for freeze-drying, oven 45 °C and oven 105 °C stabilized techniques ((**f**–**h**), respectively). Color figure available online.

Figure 2a shows the drying kinetics obtained by the 3 selected drying procedures: freeze-drying and heat treatment at 45 and 105 °C. The drying profile was similar in all cases, presenting a logarithmic trend with a greater slope in the first hours and stabilizing over time. In addition, the moisture removed was quite similar and stable after 48 h. Regarding the thermal process, the samples subjected to the highest temperature (105 °C) showed the fastest drying kinetics, achieving stability in less than 5 h. In freeze-drying and heat treatment at 45 °C, there were no significant differences in the remotion kinetics, achieving stabilization at 24 h.

Regarding the drying yield, the amount of water removed in the weight fraction from the sample was slightly higher (2%) in the case of high-temperature application. This may be due to the loss of other components (e.g., lipids) that are retained in the absorbent paper where the drying takes place, causing the freeze-drying process to achieve the lowest drying yield, as it only removes water.

The economic difference between the different treatments can be decisive In their selection. However, the quality of the flours obtained is also important; thus, it is necessary to characterize them before selecting a method as the optimal.

The first difference observed between the samples stabilized with the different treatments is their color (Figure 2b–e). These differences could be due to the degree of degradation of the samples. In this sense, the samples do not present significant differences in chroma or tone, all being located in the orange-brown zone. However, the sample treated by freeze-drying obtained higher clarity (similar to raw bagasse). This is justified by the roasting and the greater loss of water that occurs in the heat treatment, which gives a more matte color to the samples. This more tan color may be due to the generation of Maillard reactions [29]. For this reason, lower luminosity may indicate greater denaturation of the sample.

Differences were also observed after crushing and sieving the samples (Figure 2f–h). The heat treatment at 105 °C was the treatment that obtained the highest amount of fine bagasse (particle size < 420 µm). Thus, heat treatment at 105 °C obtained 71% yield, which is higher than that obtained by freeze-drying and heat treatment at 45 °C (59% and 65%, respectively). This change could be attributed to the greater brittleness of the sample after treatment at high temperatures. The high temperature breaks intermolecular bonds, which is reflected in fractionation of the molecule. In addition, this sample has fewer components that act as plasticizers (i.e., water and lipids), making it easier to grind the husk to smaller sizes [30]. Nevertheless, this remotion of plasticizers could alter internal cell components, losing active substances [31]. For this reason, the freeze-dried sample was the one with the lowest crushing yield.

The chemical composition of the different stabilized samples was obtained (Table 1). Firstly, extrapolating the dry mass ratios of the raw bagasse, a protein fraction of approximately 11.5% must be obtained, which is far from that studied by Aliyu and Bala (2011), who determined a protein fraction of 23.5% [6]. However, it is within the range obtained by Mussato (2006), who obtained a fraction of 15% [3]. This variability may be due to several factors, with the most notable being the type of malt used and its degree of modification. On the other hand, after stabilization, the crushing and sieving stages seem to increase the initially-estimated protein concentration. This could be due to those observed by Felix et al. (2018), who explained that, after sieving, the fine fraction usually contains a higher concentration of proteins [32]. Despite this, the amount of protein generated was not greater than 20%; thus it cannot be categorized as a protein concentrate according to Pearson’s classification [33]. The majority fraction of the stabilized samples are polysaccharides, whose content increases as the crushing/sieving yield increases (105 °C > 45 °C > freeze-drying). This fraction, together with the protein fraction, is the one that estimates that this material could have great potential as a raw material for the production of bioplastics, since more than 70% of the material is made up of biopolymers. Regarding the ash content, it is consistent with what was studied by Aliyu and Bala (2011), i.e., approximately between 2 and 4.5% [6]. However, the lipid content of the samples varied considerably, which is possibly associated with the hypothesis that part of the lipid fraction is absorbed in the paper used in the oven, as was mentioned before, since its content in these cases is considerably lower than that of the freeze-dried sample. Regarding the moisture remaining in the samples, it is consistent with the previously-analyzed drying kinetics, where it was observed that the drying yield was lower with freeze-drying and heat treatment at 45 °C. Finally, the fraction of other components was estimated to correspond to lignocellulosic matter with a high fiber content.

**Figure 3:**
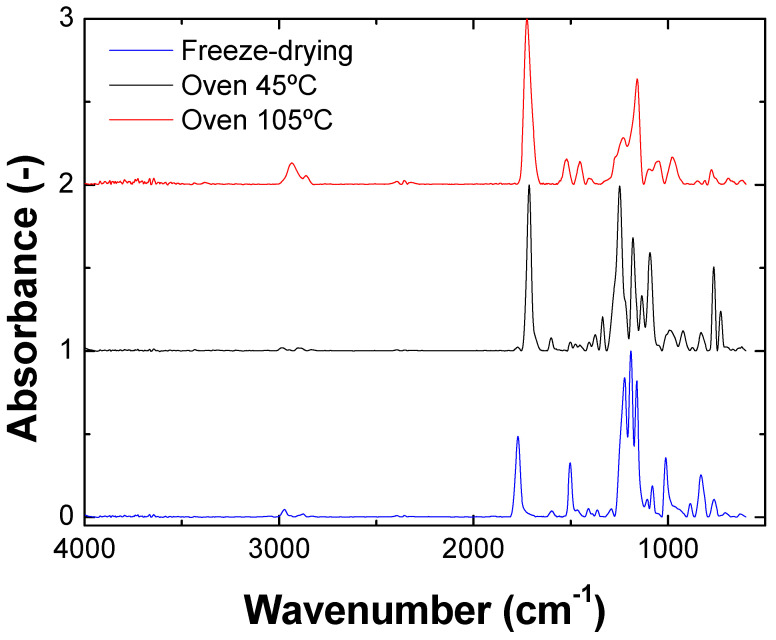
FTIR profile for stabilized bagasse by freeze-drying, oven 45 °C and oven 105 °C. Color figure available online.

The physicochemical bonds present in the different samples were evaluated by FTIR (Figure 3). The main difference between the samples is the intensity of the peaks in the 1750–1700 cm^−1^ region, corresponding to the C=O stretching of the saturated esters. This peak is associated with the hemicellulose present in the bagasse. The intensity of this peak depends on its presence in the sample, being lower in the freeze-dried sample, which had less polysaccharide content due to grinding/sieving, as was previously observed. The samples also present carboxyl and amino bonds (1800–1000 cm^−1^), which correspond to protein and polysaccharide biomolecules. A difference found in this region corresponds to amide I (1650–1630 cm^−1^), amide II (1560–1540 cm^−1^) and amide III (1460–1455 cm^−1^). In this sense, the sample that was heat-treated at 105 °C did not present the peak corresponding to amide I. In addition, the freeze-dried sample had a higher intensity in the peaks corresponding to amides II and III. The greater intensity of these peaks may be due to the lower denaturation of the sample, which did not lose its folding [34,35].

**Figure 4:**
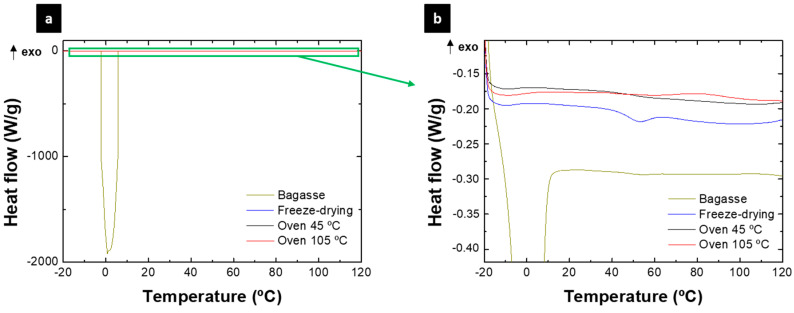
DSC profile for raw and stabilized bagasse (**a**). Zone amplification (**b**). Color figure available online.

Finally, thermal analysis was performed (Figure 4). This analysis allowed estimating the free and bound water in the samples. Free water is “freezable water”, i.e., water that is mobile and available to participate in reactions and in the deterioration of biocomposites. On the other hand, bound water is “non-freezable” water, which is strongly attached to bagasse [36]. The DSC profile of the bagasse sample shows a high endothermic peak that is associated with the high content of free water contained in the sample, which is the cause of the instability of this sample. It was observed that this area practically disappeared after treating the bagasse with the different stabilization techniques proposed. Thermogravimetric analysis (TGA) supports these results (Figure S2) since the weight loss generated by the water vaporization (approximately 50–100 °C) is lower in the case of stabilized bagasse. Thus, the sample treated at 105 °C was the one that presented the lowest weight loss, while no differences were found for the freeze-dried and the heat treatment at 45 °C. There were differences between the profiles obtained by the different stabilized samples. Firstly, the freeze-dried sample presented an endothermic peak at 50 °C, which may be associated with the denaturation of the protein fraction of the sample. In this sense, this peak was detected in the sample treated at 45 °C, although very slightly, since most of the protein was denatured during the treatment. In the sample treated at 105 °C, this peak was not identified, indicating that all the protein was denatured. This behavior is consistent with that observed by FTIR, which estimates that the least denatured sample was the freeze-dried one and the most denatured one was the heat-treated one at 105 °C. On the other hand, this analysis also allowed us to determine the glass transition of these materials, being 25, 45 and 50 °C for the samples treated by freeze-drying and heat treatment at 45 and 105 °C, respectively. This displacement of the glass transition temperature is associated with the free water content that still remains in the samples (as was observed in its chemical content), which can act as a plasticizing agent, causing this decrease in the glass transition point [37].

### 3.2. Blend

**Figure 5:**
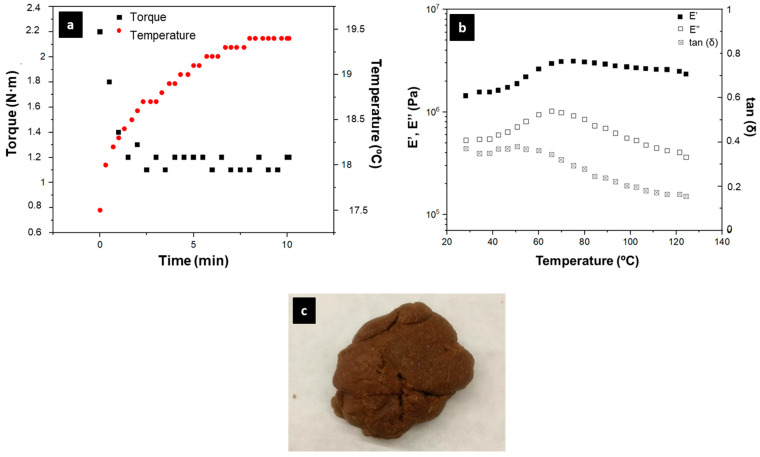
Blend characterization. Temperature and torque profile during the mixing (**a**). Elastic (E′) and viscous (E″) moduli and loss tangent (tan (δ)) in temperature ramp (**b**). Appearance of the blend (**c**).

The aim of the mixing process was to obtain a homogeneous and more processable dough-like blend. Different proportions of flour–plasticizer were studied. Nevertheless, 1:1 was the optimum ratio (Figure 5c) since, at higher plasticizer proportions, the blend was excessively wet and exuded plasticizer. On the contrary, in larger flour proportions, the blend was sandy and fell apart very easily.

Figure 5a shows the temperature and torque profile during the mixing for the blend with a 1:1 flour:plasticizer ratio. The torque underwent a first maximum peak at the beginning of the mixing, lowering its value until it stabilized. This change in torque was due to the degree of homogenization of the blend. In this way, the raw materials were more difficult to move at the beginning, since the solid (bagasse) and the liquid (glycerol) were still separated. However, they created a blend with a certain viscosity as soon as they started to homogenize, thereby facilitating the process. This degree of mixing was also observed in the temperature profile. The force necessary to move the sample was transformed into a heat (temperature) increase (i.e., this mixing process is adiabatic). However, the temperature stabilized when the sample was completely homogenized. In this way, it was observed that, after 10 min, the parameters remained stable. Therefore, 10 min of mixing was selected as the optimal time to obtain the blend, avoiding an unnecessary energy expenditure. This time was also selected as optimal in other works where similar raw materials were used [21,38].

The thermal behavior of this blend was evaluated in order to select the optimal parameters to obtain the bioplastics. Figure 5b shows the profile of elastic (E′) and viscous (E″) moduli and loss tangent (tan (δ) = E″/E′) over the temperature range studied. The value of E′ was always greater than E″, which indicates a more elastic than viscous behavior and implies a tan (δ) lower than 1 throughout the entire interval. This behavior is typical in these systems, reflecting their great solid character [39]. The thermal behavior of the blend can be divided into three zones. Firstly, E′ and E″ remain practically constant (25 to 50 °C) until reaching an inflection point (approximately 50–60 °C), which corresponds to the glass transition temperature. In fact, this transition is usually associated with a maximum of loss tangent [37]. Secondly, after the inflection point, the elastic modulus increases and the viscous modulus decreases with a significant drop in the loss tangent, probably due to a rearrangement of the structure. Finally, both modules decrease (>75 °C). This behavior may be due to the presence of starches, which improve their mechanical properties during their gelation, leading to structural changes in the samples [40,41]. Taking this behavior into account, the temperature of the cylinder was set at 50 °C to improve the fluidity of the blend towards the mold and different molding temperatures between 70 and 110 °C were studied in order to obtain the bioplastics with the best mechanical properties.

### 3.3. Bioplastics

Bioplastics presented a density of 1160 kg/m^3^ without significant differences between the different mold temperatures used. They were subjected to static tensile tests. All of them showed similar behavior with an elastic zone, where the relationship between strain and stress was linear, and breaking before showing a plastic zone (Figure S3). These results show the tenacity of the bioplastics, which withstood a certain recoverable deformation before breaking but were not capable of withstanding deformations that changed their structure. Regarding the different molding temperatures (Table 2), it was observed that the bioplastics developed at 110 °C were the ones that broke earliest (worst strain at break), while the bioplastics at 90 °C were the ones that withstood the greatest strain. These results are consistent with the study of the behavior of the blend with temperature since at 110 °C a decay of the mechanical properties of the blend was observed, while at 90 °C the values of the modules were still high. However, the bioplastics developed at 70 °C, which is the temperature at which the maximum E′ and E″ were reached in the blend temperature ramp, were not the ones with the best tensile properties. This is possibly due to the fact that starch gelation had not yet been consolidated at this temperature, meaning that the bioplastic had not yet reached its maximum structuring potential. Regarding Young’s modulus, the bioplastics developed at 110 °C presented the highest values, which indicates their great rigidity. This fact was already anticipated when trying to demold the bioplastics, resulting in the rupture of most of them. The bioplastics developed at 70 and 90 °C did not present significant differences between their Young’s modulus values. However, these values are very different from those obtained in other studies performed with protein systems, which shows that the amount of protein present in the raw material used is a critical parameter when defining the mechanical properties of the bioplastics [42,43]. In this way, the bioplastics with the highest modulus (most rigid) broke after a small elongation, while those with the lowest modulus (most flexible) underwent a greater elongation before breaking but at lower stresses. This behavior is characteristic of materials in general and polymers in particular [44]. Regarding the maximum stresses, these were very similar for all the bioplastics, being lower in the bioplastics developed at 70 °C, probably as a consequence of the lower processing temperature.

**Figure 6:**
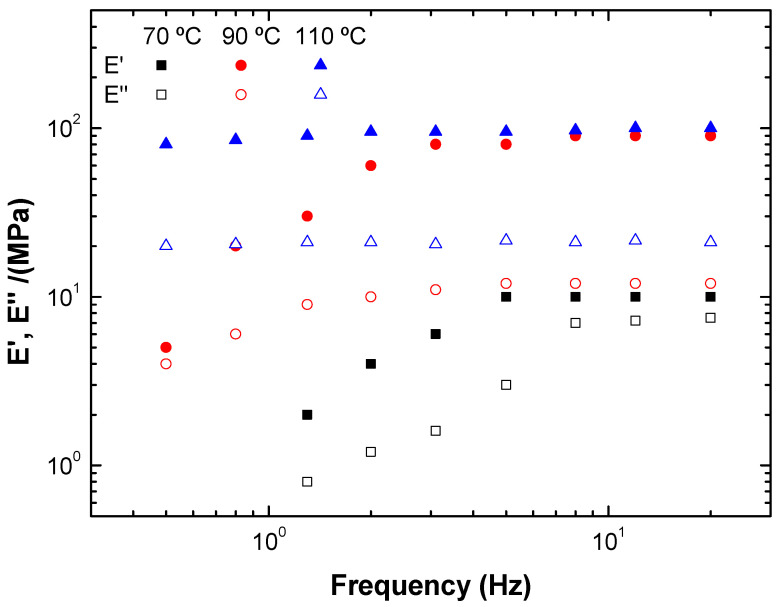
Storage (E′) and loss (E″) moduli obtained in the frequency sweep tests for the different bioplastics.

Figure 6 shows the storage (E′) and loss (E″) moduli obtained in the dynamic flexural tests throughout the entire frequency range studied. It can be observed that E′ is always found to be greater than E″ (tan (δ) < 1), which indicates the high hardness of these bioplastics. However, in all cases, E′ and E″ depended on the applied frequency, showing the low stability of the systems against bending stress. The values of the elastic modulus (E′_1_) increased with temperature, probably due to the increase in the rigidity of the systems.

Table 3 shows the water uptake capacity and soluble matter loss of the different bioplastics at different immersion times. As can be seen, the bioplastics developed at 70 °C did not withstand water immersion for more than 2 h, disintegrating in the medium and making it impossible to measure this parameter at longer times. The bioplastics processed at 90 and 110 °C endured the entire time studied (48 h), permitting the observation of differences between them. The maximum absorption was reached at 24 h for both systems, remaining stable or even losing their capacity at longer times. In addition, the bioplastics developed at 90 °C presented a greater water uptake capacity. This behavior is consistent with that obtained in the mechanical tests, since those bioplastics with better deformability were those that achieved the best water uptake capacity. These results are consistent with those found in other studies of bioplastics [21,45]. On the other hand, the soluble matter loss decreased when the mold temperature was increased from 90 to 110 °C. However, there were no significant differences with respect to immersion time, concluding that all soluble material was removed after 2 h of immersion.

**Figure 7:**
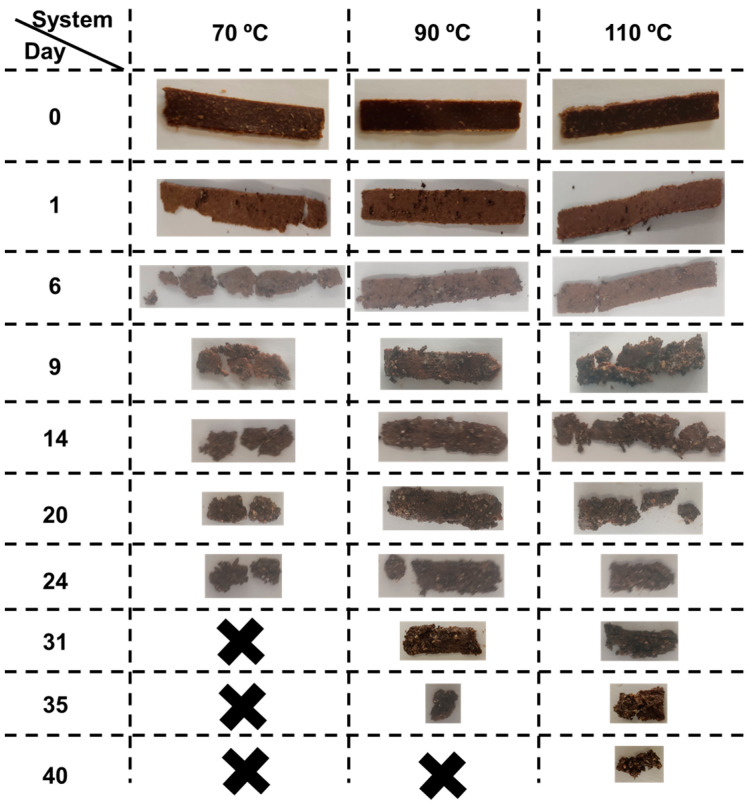
Appearance of the different bioplastics during the biodegradation tests.

Finally, the biodegradability of the bioplastics was studied (Figure 7). All bioplastics could be considered biodegradable according to the ASTM D64000 standard [46], since they decomposed in a composting medium within 6 months without releasing toxic substances. The decomposition seemed to follow the same pattern for all bioplastics, as they broke into smaller pieces until they disappeared. The maximum biodegradation time (Table 2) increased with the mold temperature. This behavior suggests that the hardest materials take the longest to decompose, which is possibly due to the fact that their partition into smaller pieces is more difficult.

## 4. Conclusions

This work demonstrates the high potential of beer bagasse as a raw material for obtaining bioactive compounds, such as proteins and polysaccharides, which can be used for the development of bioplastics. However, it is necessary to have some considerations before using it.

Firstly, industrial bagasse is a material with a large amount of free water that needs to be removed in order to stabilize the sample. Although a heat treatment may seem the most feasible method (as it is faster and cheaper), the use of freeze-drying allows for obtaining a higher quality material (less denatured). Further studies are needed to evaluate other drying methods that are more industrially feasible and that provide a similar quality compared to freeze-drying.

Regarding their use as raw materials for bioplastics, all mold temperatures allowed the production of completely biodegradable bioplastics. The lowest mold temperature (70 °C) allowed for obtaining bioplastics with less mechanical resistance that disintegrated when brought in contact with water. On the other hand, the highest mold temperature (110 °C) achieved stiffer and less flexible bioplastics. A mold temperature of 90 °C obtained bioplastics with better mechanical and absorption properties. The properties obtained in these bioplastics can be beneficial for applications such as the storage and release of water in horticulture and agriculture. The use of additives could be evaluated to improve their mechanical properties and broaden their range of applications (such as in the hygiene sector).

## Figures and Tables

**Table 1:** Chemical composition of raw bagasse and stabilized bagasse (freeze-drying, oven 45 °C and oven 105 °C). Different superscript letters in the same column mean significant differences (*p* < 0.05).

Sample	Moisture (%)	Lipids (%)	Ash (%)	Proteins (%)	Polysaccharides (%)	Others (%)
Bagasse	75.6 ± 0.4 ^a^	0.4 ± 0.1 ^c^	1.0 ± 0.1 ^c^	2.8 ± 0.2 ^c^	18.1 ± 0.5 ^d^	2.1 ± 1.5 ^b^
Freeze-drying	5.1 ± 0.1 ^b^	7.3 ± 0.1 ^a^	4.1 ± 0.2 ^b^	20.4 ± 0.8 ^a^	60.2 ± 0.8 ^c^	2.9 ± 1.3 ^ab^
Oven 45 °C	3.7 ± 0.2 ^c^	4.4 ± 0.2 ^b^	4.7 ± 0.1 ^a^	19.4 ± 0.7 ^b^	62.5 ± 0.4 ^b^	5.3 ± 1.1 ^a^
Oven 105 °C	0.8 ± 0.3 ^d^	4.4 ± 0.1 ^b^	4.7 ± 0.1 ^a^	18.4 ± 0.4 ^b^	67.4 ± 0.3 ^a^	4.3 ± 0.8 ^ab^

**Table 2:** Tensile and flexural parameters. Different superscript letters in the same column mean significant differences (*p* < 0.05).

Bioplastics	Tensile Parameters			Flexural Parameters	
	Young’s Modulus (MPa)	Maximum Stress (MPa)	Strain at Break (%)	E′_1_ (MPa)	tan δ_1_ (-)
70 °C	32 ± 20 ^b^	0.21 ± 0.02 ^b^	6.1 ± 0.5 ^b^	2 ± 1 ^c^	0.40 ± 0.05 ^a^
90 °C	37 ± 2 ^b^	0.29 ± 0.01 ^a^	8.9 ± 0.3 ^a^	30 ± 1 ^b^	0.29 ± 0.03 ^b^
110 °C	152 ± 5 ^a^	0.30 ± 0.02 ^a^	1.8 ± 0.1 ^c^	90 ± 5 ^a^	0.23 ± 0.03 ^b^

**Table 3:** Parameters obtained in the water uptake capacity and biodegradation tests for the different bioplastics. Different superscript letters (comparing water uptake capacity and soluble matter loss together and biodegradation time independently) mean significant differences (*p* < 0.05).

Bioplastics	Water Uptake Capacity (%)			Soluble Matter Loss (%)			Biodegradation Time (Days)
	2 h	24 h	48 h	2 h	24 h	48 h	
70 °C	252 ± 50 ^a^	-	-	57 ± 14 ^c^	-	-	24 ^C^
90 °C	210 ± 12 ^a^	224 ± 10 ^a^	225 ± 15 ^a^	60 ± 6 ^c^	61 ± 10 ^c^	63 ± 8 ^c^	35 ^B^
110 °C	150 ± 14 ^b^	171 ± 13 ^b^	164 ± 12 ^b^	56 ± 7 ^c^	56 ± 4 ^c^	57 ± 6 ^c^	40 ^A^

## Data Availability

Data are available upon request to the authors.

## Supplementary Materials

The following supporting information can be downloaded at: https://www.mdpi.com/article/10.3390/polym15081877/s1, Figure S1: Strain sweep tests of bioplastics processed at different mold temperature; Figure S2: Thermogravimetric (TGA) profiles of raw bagasse and bagasse stabilized by freeze-drying and thermal treatment at 45 and 105 °C; Figure S3: Stress-strain profiles of bioplastics processed at different mold temperature.
