# Supplementary material for: Stabilization and Valorization of Beer Bagasse to Obtain Bioplastics
Source: Polymers (Basel). 2023 Apr 14;15(8):1877. doi: 10.3390/polym15081877 (PMC10141695; doi:10.3390/polym15081877)
Supplement: Supplementary file 1 [file polymers-15-01877-s001.zip › polymers-2315849-supplementary.pdf]

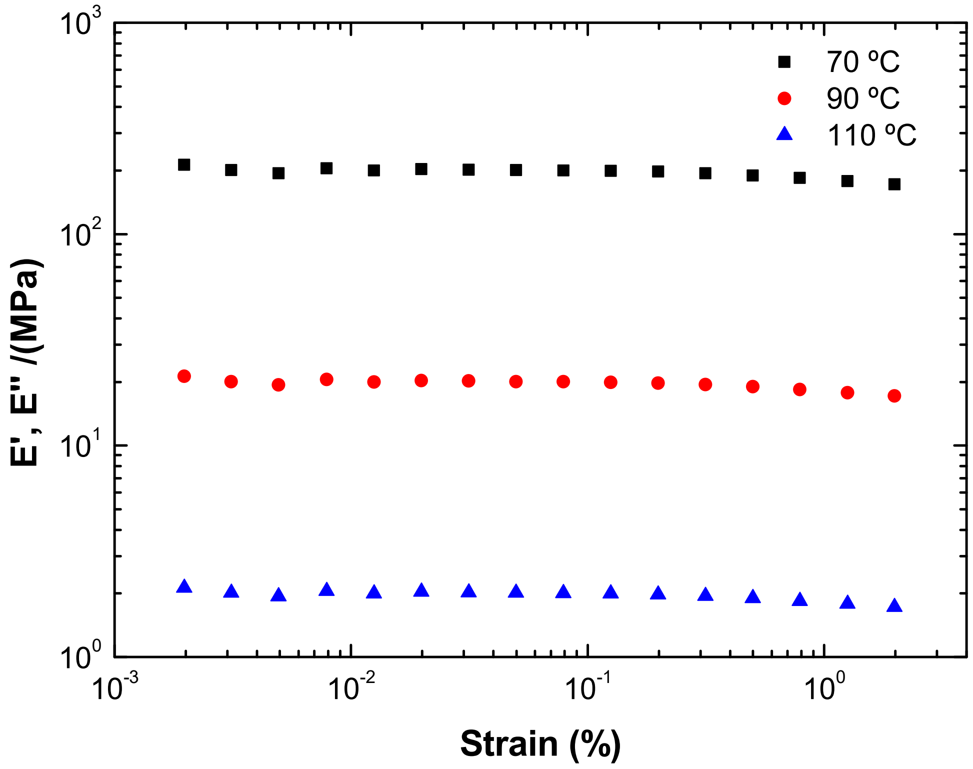

Figure S1. Strain sweep tests of bioplastics processed at different mold temperature.

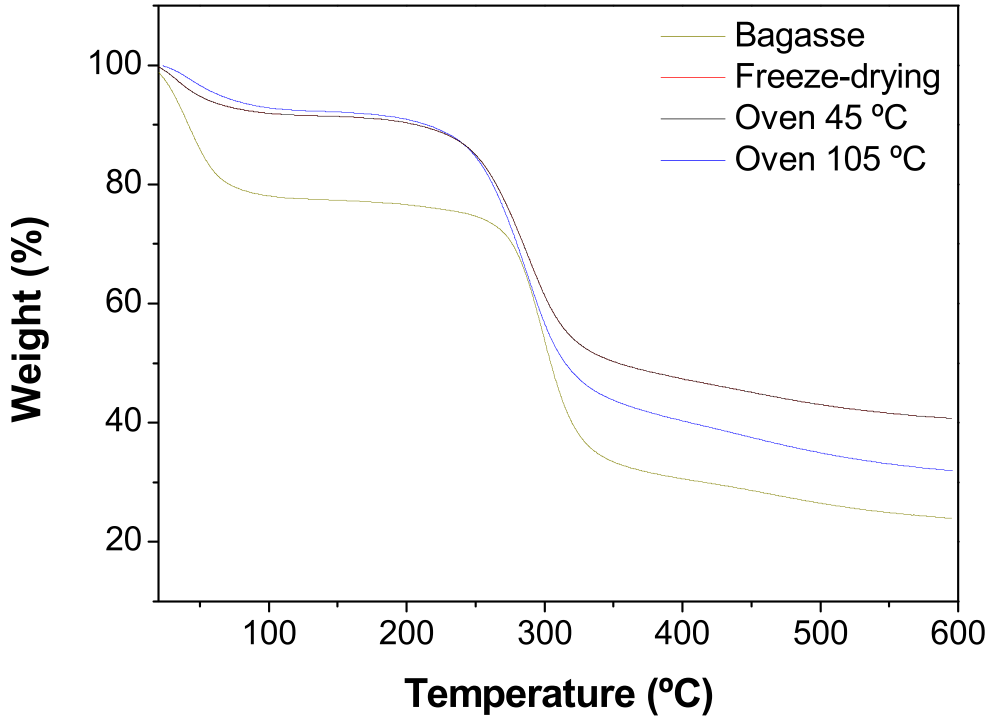

Figure S2. Thermogravimetric (TGA) profiles of raw bagasse and bagasse stabilized by freeze-drying and thermal treatment at 45 and 105 °C.

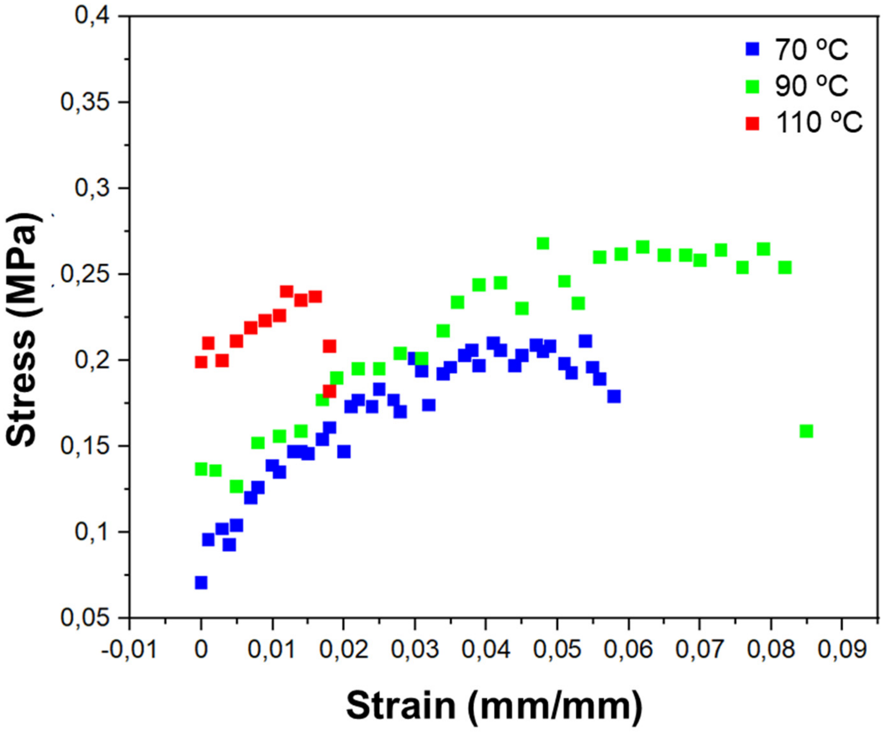

Figure S3. Stress-strain profiles of bioplastics processed at different mold temperature.
